# Review of neuroimaging in autism spectrum disorders: what have we learned and where we go from here

**DOI:** 10.1186/2040-2392-2-4

**Published:** 2011-04-18

**Authors:** Evdokia Anagnostou, Margot J Taylor

**Affiliations:** 1 Bloorview Research Institute, University of Toronto, 150 Kilgour Rd., Toronto, ON, M4G 1R8, Canada; 2 Diagnostic Imaging, Research Institute, Hospital for Sick Children, University of Toronto, 555 University Av., Toronto, Ontario, M5G 1X8, Canada

## Abstract

Autism spectrum disorder (ASD) refers to a syndrome of social communication deficits and repetitive behaviors or restrictive interests. It remains a behaviorally defined syndrome with no reliable biological markers. The goal of this review is to summarize the available neuroimaging data and examine their implication for our understanding of the neurobiology of ASD.

Although there is variability in the literature on structural magnetic resonance literature (MRI), there is evidence of volume abnormalities in both grey and white matter, with a suggestion of some region-specific differences. Early brain overgrowth is probably the most replicated finding in a subgroup of people with ASD, and new techniques, such as cortical-thickness measurements and surface morphometry have begun to elucidate in more detail the patterns of abnormalities as they evolve with age, and are implicating specific neuroanatomical or neurodevelopmental processes. Functional MRI and diffusion tensor imaging techniques suggest that such volume abnormalities are associated with atypical functional and structural connectivity in the brain, and researchers have begun to use magnetic resonance spectroscopy (MRS) techniques to explore the neurochemical substrate of such abnormalities. The data from multiple imaging methods suggests that ASD is associated with an atypically connected brain. We now need to further clarify such atypicalities, and start interpreting them in the context of what we already know about typical neurodevelopmental processes including migration and organization of the cortex. Such an approach will allow us to relate imaging findings not only to behavior, but also to genes and their expression, which may be related to such processes, and to further our understanding of the nature of neurobiologic abnormalities in ASD.

## Introduction

Autism spectrum disorders (ASD) refer to a syndrome of poor social communication abilities in combination with repetitive behaviors or restricted interests. The spectrum is usually considered to include autistic disorder, Asperger disorder, and PDD-NOS (Pervasive developmental disorder not otherwise specified), in accordance with the criteria of the fourth edition of the *Diagnostic and Statistical Manual *(DSM-IV) [1]. Recent discussions around the development of DSM-V have put forward the case that there is no evidence for consistent neurobiology differences between the three categories, and have proposed that a single spectrum disorder be included in the DSM-V. ASD remains a behaviorally defined spectrum with no known biological markers to aid with diagnosis and subgroup categorization at this point. There has been a variety of neuroimaging techniques used in attempts to understand the neurobiology of the disorder. Although great heterogeneity exists, the data from structural and functional techniques are converging to suggest underlying neuroanatomical atypicalities that affect connectivity, probably producing an inefficient system, mostly affecting complex, integrative processing.

The goal of this review is to summarize emerging themes from neuroimaging studies based on multiple imaging techniques, and to consider the implications of these results and the possible next steps that may further delineate the pathophysiology of ASD.

## Structural magnetic resonance imaging studies

When reviewing the structural and volumetric magnetic resonance imaging (MRI) data for ASD, the variability and lack of consensus in the findings seems discouraging at first, but some patterns are emerging. The most consistent finding is that of accelerated brain volume growth in early childhood, reported to be about a 10% increase in brain volume, which seems to peak around 2-4 years of age. This early overgrowth is probably followed by a plateau, although the latter has not been confirmed by large longitudinal studies [2-10]. The enlargement seems to occur in both grey and white matter, with some, but not all, studies suggesting that early in childhood there is a disproportionate contribution by white matter to this volume increase [11,12].

A crucial corollary to this question addressed in the literature deals with whether there are regional specificities to the abnormalities in brain volume. However, there is little consistency between studies. For example, abnormalities in the volume of the cerebellum have been reported since 1988. Courchesne *et al. *[13] first reported decreased volumes of vermian lobules VI and VII in the majority of their group of patients with autism. Although subsequent studies were not all able to replicate those findings, a recent meta-analysis by Stanfield *et al. *in 2007 [14] confirmed such a reduction. Similarly, abnormalities in the volume of the amygdalae have been reported in some, but not all studies [15-17]. Meta-analyses by Amaral *et al. *[10] and Stanfield *et al. *[14] suggest that age is an important factor, and that such enlargement is present only in young children with ASD. This is a crucial issue, particularly in light of the most consistent neuroanatomical difference- (the increased brain volume in young children) and is a strong argument against ignoring the age of the ASD participants in any of these analyses.

There is more consistency in volumetric studies of the corpus callosum, with multiple meta-analyses reporting decreased volume in ASD, suggesting decreased interhemispheric connectivity in this population [18,14]. Analyses of the basal ganglia have suggested increased volume of the caudate in autism [19,20], which has been correlated with severity of repetitive behaviors. The linking of the neuroanatomical findings to behavioral symptoms is vital to understanding the role of the structural changes in the aetiology of ASD. Investigations of structural measures of the cingulate have reported decreased size associated with decreased metabolic activity in ASD [21]. Volumetric abnormalities have also been reported, particularly in the frontal lobes [22] and also the temporal [23,24] and parietal lobes [25], thalamus [26], and brainstem [27].

The spread and heterogeneity of these findings suggest that autism is a widely distributed disorder affecting both grey and white matter. Newer technologies allow for the more sophisticated quantification of the structure of the grey and white matter structure. Specifically, cortical grey matter can now be described in terms of cortical thickness and surface area, the product of which produces an estimate for cortical grey-matter volume. These two measures are of particular interest. as they are hypothesized to be related to distinct neurobiological processes. Cortical thickness seems to reflect dendritic arborisation and pruning within grey matter [28], or changes in myelination at the interface of white and grey matter [29], whereas surface area varies with the degree of cortical folding or gyrification, and is thought to depend on division of progenitor cells in the periventricular area during embryogenesis [30]. Investigation of differences in these measures of cortical grey matter may provide important indications of very early neuroanatomical developmental events in the ASD population. In addition, as cortical thickness has been shown to vary during childhood, with changes occurring regionally in a developmental progression [31,32], detailed measures of cortical thickness can provide crucial information on cortical maturation and be an important index of an altered developmental trajectory in the brains of children with ASD.

Complementary studies of diffusion tensor imaging (DTI) are providing increasing insights into the structure of white matter. DTI measures water diffusion within a tissue. The tightly organized white-matter tracts restrict the diffusion of water, producing anisotropic diffusion, whereas water diffusion in the grey matter tends to be less restricted [33,34]. In DTI, the shape of diffusion is represented by an ellipsoid with three eigenvectors describing the directions of the radii, and three eigenvalues describing their length. Diffusion along the primary eigenvector represents longitudinal diffusivity, and is thought to be related to axonal integrity, a complex construct that may include accumulation of cellular debris, disordered microtubule arrangement, aggregation of microfilaments, cellular swelling and decreased axonal transport [35]. By contrast, diffusion across the two other small directions is related to radial diffusivity, and is thought to be a marker of myelin integrity, although the sensitivity of radial diffusivity to detect myelin disruption may be decreased in the case of co-occurring axonal injury [36-38]. Mean diffusivity refers to the average diffusivity along all axes, whereas the shape of the ellipsoid is represented by the calculation of fractional anisotropy (FA), a metric related to differences between the three eigenvalues. Thus, several measures can be extracted from DTI neuroimaging studies, which will contribute to a fuller understanding of differences in white-matter development in ASD.

### Cortical grey-matter studies

Hadjikhani *et al. *[39] were the first to examine cortical thickness in adults with ASD using *in vivo *MRI and in a regionally specific manner, with a vertex-by-vertex approach across the whole brain. A number of regions were found to have thinner cortex in adults with ASD relative to controls, including regions in areas known to be important for social cognition and the mirror neuron system. By contrast, Hyde *et al. *[40] found increases in cortical thickness in an autism group relative to matched controls in regions from all four lobes, in areas important for the key areas of impairment seen in autism. Increased cortical thickness was also seen in primary sensory areas, and a small number of regions had thinner cortex in the autism group.

In studies including children only, Hardan *et al. *[41] found significantly thicker cortex in the whole brain overall for boys with autism, with similar findings in the temporal and parietal lobes. In a follow-up study [18], the investigators reported that children with ASD had significant decreases in total grey matter with age relative to the control children, as well as decreases in cortical thickness with age. However, only the difference in occipital cortical thickness remained significant after adjusting for multiple comparisons, and group differences disappeared after using IQ as a covariate. By contrast, with a sample ranging in age from 10 to 65 years, Raznahan *et al. *[42] found that in regions showing an age-by-group interaction, there was no relation between age and cortical thickness in the ASD group, whereas there were decreases in cortical thickness with age in the controls. In addition, cortical thickness in typically developing children was increased relative to children with ASD at younger ages, but decreased relative to the ASD group at older ages.

Considerably fewer studies have investigated brain sulcation patterns, although atypicalities in sulcal position and depth have been reported in children and adolescents with ASD, with the most marked effects seen in the younger children [43,44]. Increased gyrification index in left frontal lobe in children but not adults, and decreased cortical folding with age in ASD relative to controls, were reported by Hardan *et al. *[45]. However, a recent study of surface area reported no age-by-group interactions for this measure [42].

Thus, most studies of cortical thickness in ASD have reported increases in values relative to typical controls, although exceptions have also been reported, with trends possibly suggesting that such differences are more pronounced in younger children, and that age-by-group interactions may be evident. In addition, there has been considerable variability in the techniques used. Advances in automated cortical surface analyses [46-48] allow fine-grained examination of regional and sub-regional differences in cortical thickness, and investigation of developmental changes in more detail. Application of these techniques may yield greater consistency in findings for comparisons of children and adults with and without ASD. Lastly, sample heterogeneity may have accounted for the heterogeneity of results as not all cohorts had been assessed with standardized diagnostic instruments (Autism Diagnostic Observation Schedule (ADOS) and Autism Diagnostic Interview, Revised (ADI-R) consistently, and some did not match for IQ, although recent findings did find an association between IQ and cortical thickness [49,50]. Studies of cortical surface are still in their infancy, but given their potential to describe differential neurodevelopmental events, hold great promise.

### DTI studies

There have been several studies examining FA in white-matter tracts in children and adults with ASD. Lower FA in a group comparison has typically been interpreted to reflect decreased organization and coherence within fibre tracts. Although the locations have varied (orbitofrontal, medial prefrontal, temporal lobe, corpus callosum, cingulate cortex, arcuate fasciculus, ILF, uncinate fasciculus, cerebellar outflow tracts, internal capsule), and different techniques have been used, including tractography and voxel-based techniques, most studies have found evidence of reduced FA in various regions in children and adults with ASD compared with control groups [51-66]. Consistent with the variable findings in other structural brain measures in this field, some recent studies have also found evidence of regions of increased FA in ASD, in samples of young children [67] and adolescents [68]. However, because FA represents the relative magnitudes of parallel and perpendicular diffusion (shape of ellipsoid), investigation of the individual eigenvalues are needed to better interpret any differences in DTI that may exist between ASD and control groups. Recent studies have begun to report the sources of these differences in radial, axial and mean diffusivity. Widespread increases in mean diffusivity have been described [69-71], suggesting a poorly organized white matter, although little consensus exists on the relative contribution of axial versus radial diffusivity in this population. In fact, abnormalities in radial diffusivity have been reported [59,60,72], implicating the myelin component of white matter in ASD pathophysiology. Despite the variability in the brain areas in which differences are reported in DTI measures between individuals with and without ASD, many groups have found evidence of atypical white-matter measures, lending credence to these findings [51-71]. However, more detailed studies analysing the more specific measures from DTI, such as radial and axial diffusivity, carefully controlling for age, may allow a more reliable picture to emerge that will further help our understanding of the aetiology of these abnormalities and the link between these changes in the brain structure and associated behavioral profiles in the ASD population.

In summary, structural studies of grey and white matter suggest an abnormal developmental trajectory of brain growth, with evidence of poorly organized white matter, increased cortical thickness and atypicalities in gyration patterns, possibly implicating abnormalities in neuronal migration, cortical organization and myelination in ASD. The abundance of inconsistent findings in the published literature on autism might reflect differences between study populations, such as age, level of impairment, and presence of medical and behavioral comorbidities in the selected groups.

## fMRI

There has been a plethora of functional neuroimaging studies in ASD, examining aspects of social cognition, language and executive functions, among others. The contribution of this method to understanding aspects of the biology of ASD has been invaluable, as it helped established that ASD is a neurological disorder (for an extensive review, see Minshew and Keller [73]).

The early fMRI studies on ASD focused on task-specific methods related to core symptom domains. The extensive literature explored patterns of activation in response to tasks related to social cognition such as face processing [74-78], theory of mind tasks [79,80], imitation [81,82], language processing such as semantic sentence comprehension [83], lexical semantic processing tasks, or tasks involving sentences of variable imagery content [84]. Repetitive behaviors have been a more difficult domain to study with scans. Cognitive paradigms used as proxies for repetitive behaviors have included executive function tasks such as those involved with inhibitory control, conflict resolution or oddball detection [85,86,73]. Other aspects of executive function, motor control and planning have also been studied, with some convergent results. For example, in a variety of tasks, atypical activation in the anterior cingulate is commonly reported in participants with ASD [87-89], suggesting a difference in ASD that can be reliably detected and can affect a range of cognitive processes.

Although a detailed review of fMRI studies in this population is beyond the scope of this review, this approach deconstructed 'unusual' behavior into recognizable neural components [73], suggested decreased cortical specialization in ASD (mild shifting of cortical location areas in response to a variety of tasks), and proposed that autism is a distributed brain systems disorder.

A series of studies followed, examining functional connectivity (Fc)MRI. Measures of FcMRI assess inter-regional temporal correlations of the blood oxygen level dependent (BOLD) signal. There are at least two approaches to conceptualizing connectivity, the more prominent of which focuses on evaluating connection strength, whereas the other examines the number of connections. Consistent with the first approach, in the original study by Just *et al. *[83], the authors found decreased functional connectivity between regions of interest activated during a language-comprehension task, and proposed that ASD may be a disorder of under-connectivity. In the many studies that followed, the concept of cortical-cortical under-connectivity has been reported with a variety of tasks related to core symptom domains, such as social cognition [90], language [91] or executive function [92] as a proxy for repetitive behaviors, and visual guided saccades and basic motor tasks [93]. The few cases of over-connectivity that have been reported, primarily affecting cortical or subcortical connections [94,95], still support the idea of atypical connectivity in ASD. The second approach was used by Noonan *et al. *[96] to study functional connectivity in the network supporting source memory, and they reported overabundant or more diffuse connectivity. The implications of these findings are that inefficient connectivity may be the hallmark of ASD, arising from both decreased signal in connections, and possibly overabundant connection between 'non-essential' regions, allowing for low-level cross talk and resulting in increased noise in the system [96]. There has also been an increased interest in studying connectivity in non-task dependent paradigms. This approach addresses the concerns that findings of under-connectivity in ASD were partially driven by activation effects, which may be confounded by task performance [97]. The presence of a default-mode network was first hypothesized in studies that identified areas of increased BOLD signal at rest compared with the active task state [98,99]. This network has now been thoroughly investigated, and includes medial structures such as the medial prefrontal cortex, anterior and posterior cingulates, medial parietal cortex and precuneus, and medial temporal regions such as the parahippocampal gyri. The resting-state network provides the opportunity to study long-distance connectivity in ASD, without complications related to task activation. Results are now emerging from such investigations with ASD, and most of the data available are consistent with decreased long-distance connectivity (frontal-posterior) [100-102] in these populations, with some evidence for increased connectivity within posterior regions. This is a relatively new area of exploration that requires significant technical development, but the early data seem consistent with other fMRI-related approaches that support the idea of poor cortical-cortical long-distance connectivity in ASD.

In summary, multiple imaging techniques based on the BOLD signal have provided evidence for decreased cortical-cortical connectivity, with possibly increased connectivity between subcortical regions and cortex, and within primary sensory areas such as the visual cortex. These results, in combination with findings of decreased cortical specialization, and supported by structural imaging studies that indicate abnormal growth and organization of both grey and white matter, reinforce the model of atypical connectivity in ASD, possibly resulting in an inefficient system with altered signal-to-noise ratio [73], that is, decreased signal with under-connectivity or increased noise with over-connectivity when defined as increased numbers of connections.

## Magnetic resonance spectroscopy

In the context of accumulating data suggesting poor structural and functional connectivity in autism, the question arises of whether techniques that provide biochemical markers of the integrity of white and grey matter, such as magnetic resonance spectroscopy (MRS) may be useful in this population. MRS allows quantification of a range of brain metabolites. The technique has been useful in other neuropsychiatric disorders, offering insights into dysfunction of grey and white matter. In addition, structural imaging and MRS studies may reflect different mechanisms of abnormal pathologies in grey and white matter, with MRS measurements being determined by the biochemical profile of underlying pathologies. Hence, MRS is a good canditate to contribute information to the study of grey and white matter in autism, which we can not possibly obtain with structural and BOLD-based techniques.

*N*-acetyl-aspartate (NAA) is the most prominent metabolite detected in the typical human brain, and is located within neurons, neuronal projections and mature oligodendrocytes. In grey matter, NAA levels are considered to reflect neuronal density, whereas in white matter, decreases in NAA are traditionally interpreted as axonal loss [103]. Decreased neuronal density in grey-matter regions that are the origin for long-range fibre tracts would result in decreased NAA in both the grey and the corresponding white matter. Of relevance to autism, a candidate gene, *AGC1 *(aspartate-glutamate carrier isoform 1), was knocked out in a mouse model [104], and the resultant profound decreased NAA levels were associated with significant myelin deficits.

When studying the white matter, other metabolites measured by MRS are also of interest, most notably myoinositol, a glial marker. There have been few pilot MRS studies performed for autism, and most have focused exclusively on the grey matter [105]. Decreased NAA seems to be the most reliable finding in the grey matter. However, this is not consistent with the explanation that early brain overgrowth is due to increased neuronal cell packing, and alternative hypotheses need to be entertained, such as reduced synaptic density, poorly differentiated cortex, column-density abnormalities and possibly inflammatory processes [106]. Only two studies have investigated white matter spectroscopy in autism. The first reported on white matter in children with ASD aged 3-4 years old, and compared them with typically developing controls and controls with developmental delay (DD), matched for age [106]. The authors found decreased NAA and myoinositol levels in children with ASD versus typical controls, whereas no differences were noted compared with the DD group. A second study reported no differences in NAA in the centrum semiovale in children with ASD versus typical controls, although no absolute quantification was carried out [107].

Researchers have also started to use MRS for examining theories related to excitation and inhibition ratios in ASD [108]. Such theories arise from imaging work that has been previously reviewed (suggesting inefficient connectivity in brain circuits) and the high prevalence of epilepsy in individuals with ASD (suggesting an increased excitation to inhibition ratio), as well as candidate genes involved in excitatory and inhibitory synaptic function and animal models [109]. Given that such theories have obvious implications for the study of connectivity, the ability to examine glutamate, glutamine and γ-amino butyric acid (GABA) properly holds promise. Early studies have presented some inconsistent findings [110,111], but technical limitations in this area, as well as issues related to possible developmental effects in the maturation of the glutamate system, have rendered progress particularly slow.

In summary, magnetic resonance spectroscopy techniques have the potential of elucidating the chemical substrate of aberrant connectivity in autism and as such hold promise to ultimately assist with the development of biologically guided treatments.

## Future developments

Currently, structural and functional imaging studies are converging on the hypothesis that ASD is associated with atypical connectivity, probably producing a system that is ineffective for more complex information processing. Although this in itself has been argued to be a paradigm shift in our assumptions about the neurobiology of the disorder, we still need to understand this phenomenon within the context of developmental neurobiology. As we know from typical neurodevelopment, there is significant plasticity in the brain, with connections being made and retracted in response to activity [112]. Thus, functional connectivity is a dynamic, constantly evolving process; the brain is shaped by experience (for an excellent review paper discussing these issues, see Muller [97]). In addition, grey and white matter are not separate compartments; white matter is composed of the myelinated axons of the neuronal somata that exist within the cortical layers, and they need to be studied concurrently. Local cortical architecture and connectivity are linked processes, and are the result of migration and organization/differentiation, which are part of the same set of developmental processes. Previously described abnormalities in both gyrification index and cortical thickness in individuals with ASD suggest that both processes are involved in the pathophysiology of the disorder. This is consistent with neuropathology and animal model studies that have described abnormalities in cytoarchitecture and in minicolumns, and altered excitation:inhibition ratio in ASD. The implications of seeing locally differentiated architecture and connectivity as the result of linked developmental processes has implications for future studies, and for the integration of imaging with genetics and phenotype studies to further elucidate the pathophysiology of the disorder. Specifically, variations or mutations in candidate genes related to molecular pathways linking synaptic signals to gene expression and protein synthesis (for example, tuberous sclerosis protein (TSC)1 and 2, phosphatase and tensin homolog (PTEN)), translation and protein stability (for example, fragile X mental retardation 1 (FMR1), ubiquitin protein ligase E3A (UBLE3A)), development of neuronal processes, synapses and axon tracts (neuroligins, protocadherin 10, contactin 3 and 4, SHANK3), and myelin integrity-related genes could be studied in individuals who carry them. This could help elucidate the effect of the molecular alterations seen in ASD on circuitry integrity and function and increase our understanding of the shared pathophysiology of this heterogeneous disorder. Understanding the neurodevelopmental processes that are linked to the neuroimaging findings seen in ASD could potentially make available a list of other candidate genes involved in migration and organization, and progress in this area is already evident. As a result, we have the opportunity to close the loop from genes and gene expression to brain architecture and function to behavior. It is hoped that such an understanding will lead to neurobiologically informed interventions.

Given the developmental nature of this disorder and the observed heterogeneity, studies with larger samples and longitudinal studies are required to adequately describe the variance in ASD and elucidate brain behavior relationships. In addition, complementary methods may be required to understand patterns of growth, including studies of cortical thickness versus surface morphometry, especially as they have the potential to affect different, although linked developmental processes. Using newer automated techniques along with careful subject characterization is likely to decrease the variance seen in existing studies. Studies of structural connectivity (DTI) are of great interest, especially when combined with studies of spatial and temporal dynamics of brain activity in specific circuits and contrasted to resting networks (fMRI, magnetoencephalography) to further elucidate the nature of connectivity abnormalities in the brain. Exploration of the excitation:inhibition ratio using neurochemical techniques (MRS sequences exploring developmental trajectory of glutamate and γ-aminobutyric acid (GABA) maturation) has the potential to further clarify the construct of altered signal-to-noise ratio in this population. Combining some of these methods within the same cohort of subjects and in a developmental manner should be a major focus for future works, as this has the potential to shed light in the nature of shared developmental abnormalities in ASD and to link imaging findings to underlying molecular neurobiology, a necessary step to further facilitate experimental therapeutics.

## Competing interests

Evdokia Anagnostou has been a consultant on a fees-free basis to Neuropharm and Proximagen. Margot J. Taylor has no competing interests to disclose.

## Authors' contributions

EA contributed to the conception and design of this review and drafted most the manuscript. MJT contributed to the design of this review and helped to draft the manuscript. All authors have read and approved the final manuscript.
